# Major Depression in Postpartum Women during the COVID-19 Pandemic: Can Social Support Buffer Psychosocial Risks and Substance Use?

**DOI:** 10.3390/ijerph192315748

**Published:** 2022-11-26

**Authors:** Krista J. Howard, Caleb Leong, Sidney Chambless, Timothy J. Grigsby, Millie Cordaro, Jessica K. Perrotte, Jeffrey T. Howard

**Affiliations:** 1 Department of Psychology, Texas State University, 601 University Dr., San Marcos, TX 78666, USA; 2 Department of Public Health, University of Texas at San Antonio, One UTSA Circle, San Antonio, TX 78249, USA; 3 Consequences of Trauma Working Group, The Center for Community-Based and Applied Health Research, University of Texas at San Antonio, One UTSA Circle, San Antonio, TX 78249, USA; 4 Department of Social and Behavioral Health, University of Nevada, Las Vegas, NV 89119, USA

**Keywords:** postpartum, pregnancy, depression, substance use, social support

## Abstract

Rates of mood disorders and substance use increased during the COVID-19 pandemic for postpartum women. The present study’s aims were to: (1) examine the prevalence of major depressive disorder (MDD) in postpartum women during the COVID-19 pandemic, and (2) evaluate whether social support can buffer the associations between MDD, psychosocial factors (perceived stress, generalized anxiety, and intimate partner violence) and substance use (alcohol and drug use). A nationwide survey included 593 postpartum mothers (within 12 months from birth). Participants were assessed for a provisional diagnosis of MDD, and provided responses on validated instruments measuring stress, intimate partner violence, suicidal ideation, generalized anxiety, social support, and substance use. A hierarchical logistic regression model assessed the association of psychosocial factors and substance use with MDD. The final model shows that social support attenuates the association of MDD with perceived stress, alcohol use, and drug use, but does not buffer the relationship of MDD with anxiety or intimate partner violence. Social support was shown to significantly attenuate the effects of stress, alcohol use, and drug use on MDD, suggesting that the presence of a strong, supportive social network should be an area of increased focus for public health and healthcare professionals when caring for postpartum women.

## 1. Introduction

New mothers face significant challenges during the postpartum period, which can increase the risk for emotional dysregulation and psychopathology [1,2]. Postpartum mental health issues are a common complication following pregnancy. Approximately 10–15% of mothers are affected with postpartum depression and up to half of those affected will have symptoms lasting for more than six months [3]. Further, postpartum mental health issues contribute to approximately 9% of pregnancy-related deaths [4]. While postpartum depression varies in severity, it can include expressions of intense fatigue or sadness, social isolation, lack of concentration, and suicidal ideation, and often results in poor sleep, irritability, and somatic complaints [5].

Mental health issues and substance use behaviors have increased since the COVID-19 pandemic began, not only in the general population, but for pregnant and recently pregnant women as well. In the United States (U.S.), the prevalence of adults meeting clinical criteria for major depressive disorder in April 2020 was 22.7% [6] compared to the 10.4% pre-pandemic 12-month estimates of major depressive disorder [7]. Similarly, the percentage of U.S. adults who met the provisional criteria for generalized anxiety disorder during April 2020 was 17.9% [8] compared to 1.8% pre-pandemic 12-month estimates [9]. A meta-analysis of postpartum depression during the pandemic identified rates ranging between 12% and 44%, and estimates vary based upon the type of assessment used and the time since delivery [10]. Beyond noted increases in substance use frequency [11,12], alcohol-related deaths [13] and drug-related overdoses [14] also increased during the pandemic.

Recent studies showed that trends in alcohol and drug-related mortality for pregnant and recently pregnant women have increased significantly from 2015 through 2019 [15], and found that rates of binge drinking and heavy alcohol consumption for pregnant women have significantly increased from 2011 through 2020 [16]. The COVID-19 pandemic, an ongoing traumatic stressor event, has put postpartum women at heightened risk for developing both mental health disorders [17] and substance use problems.

Postpartum women face stressful physiological and emotional changes, which have been exacerbated by the COVID-19 pandemic’s disruptive impact on access to postnatal care, isolation, and pandemic uncertainty [18]. Isolation and uncertainty during the COVID-19 pandemic yielded high rates of psychological distress in adults, including increases in panic and anxiety disorders, and increased depression symptomology [19]. Self-harm ideation is prevalent among mothers with postpartum depression both before and during the COVID-19 pandemic [20,21]. Heightened psychological distress in postpartum women can be linked to increased risk for substance use, a maladaptive coping mechanism [22]. Maternal substance use, including both alcohol and drug use, increased in 39.2% of mothers of children ages 0–8 years-old since the onset of COVID-19 [23]. A study in New York investigated substance use for pregnant and mothers who had given birth in the prior 12 months between April and June 2020, revealing high prevalence rates of substance use for tobacco (20.7%), cannabis (15.0%), and alcohol use (38.0%) [18]. These rates are high in comparison to the prevalence rates from a study conducted in 2017 which showed lower rates for tobacco (14.7%), cannabis (7.1%), and alcohol use (11.5%) among pregnant women [22]. Further, opioid use during pregnancy is associated with an increased risk for postpartum drug overdose or death [24]. Depressive disorders and substance use/abuse are often comorbid and are co-occurring risk factors for each other [25]; this comorbidity yields higher risks for suicide [26].

Substance use during and following pregnancy is frequently interconnected with many other factors, including intimate partner violence and lack of social support [27]. Reports of intimate partner violence and domestic violence spiked since the onset of the COVID-19 pandemic. Researchers in the U.S. note that intimate partner violence during the COVID-19 pandemic may be underreported, as victims have had greater difficulty in distancing themselves from abusive partners [28].

Research has established a buffering effect as a key mechanism of social support systems for reducing the psychological impact of stressful environments [29,30]. Social support may act as a protective factor for pregnant women experiencing stressful environments and could be helpful as an intervention tool to improve perinatal mental health [29]. Further, pregnant and postpartum women view social support as a crucial aspect for obtaining positive substance use treatment outcomes [31]. Pregnant and postpartum women with a substance abuse disorder along with a strong social support system demonstrate enhanced resilience and an increased likelihood of sustained recovery [32].

Because the trends for mortality related to increased alcohol and substance misuse have increased for pregnant and postpartum women since before the pandemic, and the rates of mood disorders and substance use have been elevated during the COVID-19 pandemic for the general population and postpartum women, the aims of this study were two-fold. The first aim was to examine the overall prevalence of major depressive disorder (MDD) in a nationwide sample of postpartum women during the COVID-19 pandemic. The second aim was to evaluate the associations between MDD, psychosocial factors (perceived stress, generalized anxiety, and intimate partner violence) and substance use (alcohol and drug use), and to test if those associations are attenuated by social support.

## 2. Materials and Methods

### 2.1. Study Design

This study utilized a cross-section, national survey of pregnant and postpartum women aged 18 and older. The study was reviewed by the University of Texas at San Antonio Institutional Review Board and was determined to be research not involving human subjects as defined in 45 CFR 46.104(3)(A).

### 2.2. Participants

The participants in this study included both currently pregnant and postpartum (defined as having given birth within one year from survey date) women recruited and incentivized through national panels administered by Qualtrics. A total of 18,900 individuals were sent a survey invitation, and 2739 (14.4%) participants clicked the link to take the survey. Of the 2739 participants who clicked the survey link, 938 (34.2%) were excluded from participation due to demographic strata quotas to ensure adequate sampling of racial/ethnic minorities already being met. In order to obtain a sample with similar racial/ethnic composition as the total US population of birth mothers, we used a 50% quota limit for non-Hispanic white women, which matches the total percentage of non-Hispanic white birth mothers. This process was successful in producing a racial/ethnic composition similar to the total US population of birth mothers, with 50% being non-Hispanic white and 50% be non-white mothers (Table 1). In addition, 469 (17.1%) were excluded for refusing to provide consent or indicating they did not meet the current or recently pregnant criterion, 52 (1.9%) were excluded due to data quality checks, and 1280 (46.7%) participants completed the survey (679, 53% pregnant; 601, 47% recently pregnant). The present study is based on the 601 postpartum women who completed the survey, of which 593 participants completed the PHQ-9 measure that was used to assess and provide the provisional diagnosis of Major Depressive Disorder (MDD) and are included in the current study (98.7% completion response rate). The mean age of the postpartum participants at the time they completed the survey was 35.2 (SD = 10.1) years, 49.9% were non-Hispanic White, and 17.4% were Hispanic/Latina.

### 2.3. Measures

Only self-reported biological females completed the survey. Participants were also asked for basic demographics including age, race/ethnicity, marital status, education level, type of healthcare coverage, and current employment status.

#### 2.3.1. Major Depressive Disorder (PHQ-9)

The Patient Health Questionnaire—Major Depressive Disorder (PHQ-9) subscale was used to assess whether the participants meet the criteria for a provisional diagnosis of Major Depressive Disorder [33]. The Major Depressive Disorder (MDD) subscale of the PHQ includes 9 items measured on a 4-point Likert scale from 0 = not at all to 3 = nearly every day evaluating experiences in past two weeks, such as “Little interest or pleasure in doing things”. To meet the criteria for a provisional diagnosis of MDD, a cut-off score of 10 was use [34]. For the current study, the PHQ-9 had good internal consistency (Cronbach’s alpha = 0.92; M = 12.5, SD = 7.6).

#### 2.3.2. Suicidal Ideation/Self-Harm

The Patient Health Questionnaire (PHQ-9) single-item #9 was used to assess suicidal ideation. Item #9 from the PHQ-9 provides the prompt, “Over the last 2 weeks, how often have you… (had) thoughts that you would be better off dead or of hurting yourself in some way?” The participants are asked to respond using the options: not at all, several days, more than half the days, and nearly every day. Item 9 from the PHQ has been shown to be effective in predicting suicidal ideation [35].

#### 2.3.3. Generalized Anxiety Disorder (GAD-7)

The Patient Health Questionnaire—Generalized Anxiety Disorder (GAD-7) subscale was used for assessing the presence of Generalized Anxiety Disorder (GAD). The GAD-7 subscale includes 7 items on a 3-point Likert scale ranging from 0 = not at all to 3 = nearly every day that evaluates the extent to which the individual has been bothered by specifically stated problems during the last four weeks. A cut-off score of 10 is used to provide a provisional diagnosis of GAD (Spitzer et al., 2006). An example of an item from the GAD subscale is, “Feeling restless so that it is hard to sit still.” The GAD-7 had good internal consistency in our sample (Cronbach’s alpha = 0.86; M = 9.5, SD = 6.0).

#### 2.3.4. Interpersonal Support Evaluation List (ISEL-12)

The ISEL-12 is a measure of general social support [36]. This questionnaire includes 12 items each measured on a 4-point Likert scale, with responses ranging from 1 = Definitely False to 4 = Definitely True. An example of an item on this scale is, “When I need suggestions on how to deal with a personal problem, I know someone I can turn to.” For this sample, the overall ISEL-12 scale (M = 32.3, SD = 6.6) achieved an alpha reliability of 0.78.

#### 2.3.5. Perceived Stress Scale (PSS)

The PSS is a highly utilized 10-item self-report measure that assesses general stress over the past four weeks using a Likert scale from 0 = never to 4 = very often [37]. Scores range from 0 to 40, with higher scores indicating a greater degree of perceived stress. An example item from the PSS is, “In the past month, how often have you been nervous or stressed?” The present study’s Cronbach’s alpha was = 0.65 (M = 20.2, SD = 5.6).

#### 2.3.6. Colorado Behavioral Risk Factor Surveillance System (BRFSS) Violence Screener

The Colorado BRFSS Violence Screener is a well-validated 3-item self-report measure that assesses exposure to verbal or physical violence in the past year [38]. Each item is measured as a yes/no response, and any positive response is considered a positive screen for violence exposure. Specific items include questions like “… on any occasion were you hit, slapped, kicked, raped, or otherwise physically hurt by someone you know or knew intimately, such as a spouse, partner, ex-spouse or partner, boyfriend, girlfriend, or date?”.

#### 2.3.7. Alcohol Use Disorders Identification Test (AUDIT)

The AUDIT is a well-validated 10-item scale that assesses risky and harmful alcohol consumption behavior over the past year [39]. The AUDIT includes questions that measure the amount and frequency of alcohol intake, alcohol dependence, and problems related to alcohol consumption, and has a total score range of 0 to 40 [40]. Scores over 8 are considered to be harmful consumption, with score range categories of abstainer (0), ‘low-risk consumption’ (1–7), ‘harmful consumption’ (8–14) and ‘likely moderate to severe alcohol use disorder’ (>14) [37]. For this study’s sample, the Cronbach’s alpha was 0.94 (M = 9.1, SD = 9.7).

#### 2.3.8. Drug Abuse Screening Test (DAST-20)

The DAST-20 is a well-validated 20-item scale assessing the extent of drug-related problems and consequences [41,42]. Items in the scale are yes/no questions, such as “Have you used drugs other than those required for medical reasons?” Summed responses create a total score that ranges from 0 to 20, with score range risk categories of ‘none’ (0), ‘low’ (1–5), ‘moderate’ (6–10), ‘substantial’ (11–15) and ‘severe’ (16–20) [42]. For this study’s sample, the Cronbach’s alpha was 0.92 (M = 4.8, SD = 5.3).

### 2.4. Statistical Analysis

Univariate comparisons were conducted using either Independent *t*-tests or Mann–Whitney U tests with Cohen’s d effect size estimates for comparisons of continuous variables and Chi-square tests of Independence or Fisher Exact tests with Contingency Coefficients for effect size estimates for comparisons of categorical variables. For all univariate tests, two-tailed tests were conducted using a *p*-value of 0.05 for significance. Pairwise deletion was used for missing data points or extraneous values. Because the univariate comparisons were conducted to determine the variables to be included in the multivariate test, adjustments for multiple comparisons were not conducted.

A hierarchical binary logistic regression was conducted to determine (Block 2) which psychosocial and substance use factors were significantly associated with MDD, while controlling for covariates (Block 1). Next, Block 3 was included to determine if the ISEL-12 (social support measure) would attenuate associations between MDD and the psychosocial or substance use factors from Block 2. First-order interaction terms between ISEL-12, and psychosocial and substance use factors were examined to test whether associations between psychosocial and substance use factors are modified by ISEL-12. Results are reported as odds ratios (OR) with 95% confidence intervals (CI), and *p*-values. All analyses were conducted using SPSS version 27 (IBM, Inc., Chicago, IL, USA).

## 3. Results

### 3.1. Descriptive Analysis

There were 593 self-identified, biological women included in the study who had been pregnant within the past 12 months but were not currently pregnant. Of the 593 participants, 323 (54.5%) met the criterion for a provisional MDD diagnosis. (Table 1). A significant difference in mean age was identified between the two comparison groups, such that women with MDD were younger than women without MDD (*p* < 0.001). Differences in racial/ethnic distribution were also noted, with a higher proportion of Blacks and Hispanic/Latino/a participants meeting the criteria for MDD, and a greater proportion of Whites without MDD (*p* = 0.021). Women with MDD were less likely to be married than women without MDD (*p* = 0.023). No differences in education levels were identified between those with MDD and without MDD (*p* > 0.05). Most women were not first-time mothers, and the percentage of first-time mothers did not differ between women with MDD and without MDD (*p* = 0.575). However, this may have contributed to a higher mean age in our sample. A higher percentage of women with MDD reported working full-time or part-time, and a lower percentage reported being a student/retired/disabled or being a stay-at-home-mom compared to women without MDD (*p* = 0.04). Women with MDD were less likely to have healthcare coverage through their employer or through private-pay or healthcare.gov, and more likely to have healthcare coverage through Medicaid, Medicare, or to have no coverage at all (*p* = 0.015).

Psychosocial measures differed significantly between postpartum women with and without MDD. (Table 2). The prevalence of Generalized Anxiety Disorder (GAD) differed significantly such that 61.1% of women with MDD also had GAD compared to only 9.3% among women without MDD (*p* < 0.001). Suicidal ideation, based on item 9 from the PHQ-9, was higher in women with MDD, where 19.2% and 37.2% reported suicidal ideation nearly every day and more than half the days, respectively, compared to 0.7% and 0.7% for women without MDD (*p* < 0.001).

Perceived stress also differed significantly, such that the mean score on the PSS higher for women with MDD compared to women without MDD (*p* < 0.001). The prevalence of a positive violence screen was significantly higher (52.0%) for women with MDD compared to 17.0% for women without MDD (*p* < 0.001). Mean scores on the ISEL-12 social support measure were significantly lower for women with MDD compared to women without MDD (*p* < 0.001).

Alcohol and substance use behavior was also significantly higher among postpartum women with MDD compared to women without MDD. (Table 3). Comparisons of alcohol misuse showed that the mean AUDIT score was significantly higher for women with MDD compared to women without MDD (*p* < 0.001). Among women with MDD, 36.3% were considered alcohol dependent (likely moderate-severe alcohol use disorder) and 18.3% were considered to have harmful alcohol consumption, compared to 8.4% and 10.5%, respectively, for women without MDD (*p* < 0.001). Similarly, comparisons of drug use showed that the mean DAST score was significantly higher for postpartum women with MDD compared to those without MDD (*p* < 0.001).

### 3.2. Multivariate Hierarchical Logistic Regression Results

Results of the hierarchical, multivariable logistic regression analysis are presented in Table 4. In Block 1, older age was associated with lower odds of MDD (OR = 0.969; 95% CI: 0.949, 0.989; *p* = 0.003), but race/ethnicity, marital status and employment status were not significantly associated with MDD.

In Block 2, the provisional diagnosis of GAD was associated with more than a five-fold increase in the odds of MDD (OR = 5.692; 95% CI: 3.068, 10.562; *p* < 0.001). Each unit increase in the PSS was associated with a 23.8% increase in the odds of MDD (OR = 1.238; 95% CI: 1.156, 1.326; *p* < 0.001). A positive violence screen was associated with a 118.4% increase in the odds of MDD (OR = 2.184; 95% CI: 1.166, 4.091; *p* = 0.015). Each one-unit increase in the AUDIT and DAST scores were associated with 6.3% (OR = 1.063; 95% CI: 1.018, 1.109; *p* = 0.006) and 10.6% (OR = 1.106; 95% CI: 1.028, 1.190; *p* = 0.007) increase in the odds of MDD, respectively. Age, race/ethnicity, marital status, and employment status were not significant in Block 2.

In Block 3, the ISEL-12 social support score was significant, and each one-unit increase in the ISEL-12 was associated with a 7.4% decrease in the odds of MDD (OR = 0.926; 95% CI: 0.884, 0.970; *p* = 0.001). The provisional diagnosis of GAD (OR = 5.694; 95% CI: 3.024, 10.725; *p* < 0.001) and a positive violence screen (OR = 2.278; 95% CI: 1.201, 4.321; *p* = 0.012) remained significant, and their associations were not attenuated with the inclusion of the ISEL-12 social support scale. The associations for PSS (OR = 1.205; 95% CI: 1.124, 1.292; *p* < 0.001), AUDIT (OR = 1.050; 95% CI: 1.005, 1.096; *p* = 0.029) and the DAST (OR = 1.088; 95% CI: 1.012, 1.171; *p* = 0.023) with MDD were all significant, but were attenuated by 13.9%, 20.6% and 17.0%, respectively. First-order interactions between ISEL-12 and PSS, ISEL-12 and AUDIT, and ISEL-12 and DAST were tested, but were not statistically significant. Employment status (OR = 1.899; 95% CI: 1.009, 3.574; *p* = 0.047) was also statistically significant in Block 3, but age, race/ethnicity and marital status were not significant.

## 4. Discussion

In this study, over half (54.5%) of the postpartum sample met the criteria for a provisional diagnosis of MDD. The results also identified that multiple psychosocial variables were associated with MDD, including GAD, increased perceived stress (PSS), a positive violence screen (Colorado BRFSS Violence Screener), high hazardous alcohol use scores (AUDIT), and high drug abuse scores (DAST). Further, the results suggest that social support plays an important role in MDD among postpartum women. Lower scores on social support were significantly associated with higher odds of MDD. Additionally, social support was shown to buffer the effects of perceived stress, alcohol abuse, and drug abuse, but not the effects of anxiety and violence exposure, on MDD among postpartum women.

### 4.1. Prevalence of MDD

To our knowledge, our study is the first to use the PHQ-9 to assess MDD and estimate its prevalence among postpartum women during the COVID-19 pandemic era. The MDD prevalence estimate in our sample of 54.5% is higher than most previously reported studies performed during the COVID-19 pandemic. Previous studies of postpartum depression in the U.S. using the Edinburgh Postpartum Depression Scale (EPDS) reported prevalence of probable MDD of 12.4% (Silverman et al., 2020), 11% [43], 14.3% [44], and 38% [20] in postpartum women. A recent meta-analyses of non-U.S. studies reported prevalence estimates of 34% [45] and 28% [9] in postpartum women during the COVID-19 pandemic. In validation studies, the PHQ-9 and EPDS instruments were highly correlated, with high and statistically indistinguishable area under the curve, and similar sensitivity and specificity in predicting major depressive events [46]. However, the recall period is longer for PHQ-9 than EPDS, with different clinical cut-off criteria for assessing provisional diagnosis of MDD. This could partially explain why our estimates are higher than previous postpartum depression estimates. Given that individuals with MDD are the most affected by suicidal ideation, and suicidal ideation is a precondition for suicide attempts, it is critical to assess postpartum women with MDD symptoms for suicidality.

### 4.2. Social Support Attenuates the Effects of Stress, Hazardous Alcohol Use & Drug Abuse

The results of the present study indicate that close to 20% of the variance in alcohol-MDD and drug abuse-MDD associations were explained by perceived social support. This finding suggests a potential pathway of protection against alcohol and drug abuse and MDD through improved social support, which warrants further attention in postpartum women. As research has shown that the postpartum period is already a stressful time for the mother [1,2], adequate social support is a crucial component of safeguarding the health and wellbeing of new mothers [3]. Prior studies have shown that self-medication with alcohol and drugs are frequently used to cope with mood and anxiety disorders [47], which may be more pronounced for postpartum women. For new mothers who lack social support during the postpartum period, self-medicating with alcohol and/or drugs may provide a source of comfort, security, and stress-reduction [48]. Having a strong social support network may buffer against the stress and responsibility of caring for a newborn and increase levels of perceived maternal self-efficacy [49]. This can further reduce the likelihood of a postpartum mother turning to alcohol and drugs, and complications associated with alcohol- and drug-related overdose, including death [13,14,15]. In addition to the value of general social support, psychosocial interventions have been effective in reducing alcohol consumption and increasing abstinence rates in pregnant and postpartum women [50].

### 4.3. Social Support Does Not Attenuate the Effects of Anxiety & Violence Exposure

While social support showed a significant attenuating effect on the impact of perceived stress, alcohol abuse, and drug abuse on MDD, we did not observe a relationship between social support and anxiety with MDD. This finding is in line with previous longitudinal research [51] and suggests that for recently postpartum women who met the criteria for GAD, the presence of social support did not significantly reduce the impact of anxiety on MDD. Moreover, research has shown that individuals with pre-pandemic mental health disorders experienced little benefit from high levels of social support regarding GAD symptomology [52]. We did not assess timing of social support which might be important in predicting mental health status among postpartum women [53,54]. Future longitudinal research is needed to understand the complexities of timing, type, and source of social support as it relates to postpartum mental health.

Social support also did not attenuate the association between violence exposure and MDD, suggesting that having high levels of perceived social support is not enough to counteract the harmful consequences of living in a violent or abusive home environment. The effects of some forms of intimate partner violence on postpartum depression are more pronounced than others [55]. In addition, previous research has shown the buffering effect of social support is strongest with lower levels of abuse [56]. Others [28] recognized that during the COVID-19 pandemic, the number of calls to domestic-abuse support hotlines dropped dramatically, which was attributed to victims being unable to safely access support networks. More direct measures should be taken by community members, public health professionals, and law enforcement to ensure the physical safety of new mothers and their babies, especially during the postpartum period.

### 4.4. Strengths and Limitations

The present study is not without limitations. First, the data are based on self-reported psychosocial and behavioral measures, which are susceptible to potential recall bias. Second, some questions focused on sensitive topics, such as alcohol and drug use and exposure to violence, which may also be influenced by social desirability bias [57]. Third, the data were collected cross-sectionally in the U.S. during April 2022, thus the results should be interpreted as a snapshot of the experience of postpartum women in the U.S. during this specific time frame. Finally, the data collected in our survey included postpartum women who self-selected to participate in our study, and thus may not be representative of all postpartum women. The mean age of our sample was slightly older than the overall US mean age for mothers at birth as of 2019, but consistent with a long-term trend of increase age at birth for mothers in the US [58].

## 5. Conclusions

We found a significant relationship between psychosocial stressors, social support, and MDD among postpartum women in the U.S. during the COVID-19 pandemic. Anxiety, perceived stress, violence exposure, alcohol abuse, and drug abuse were all shown to increase the odds of MDD. Furthermore, social support was shown to significantly attenuate the effects of stress, alcohol abuse, and drug abuse on MDD, suggesting that the presence of a strong, supportive social network should be an area of increased focus for public health and healthcare professionals when caring for recently pregnant women. Finally, as social support was not shown to significantly attenuate the effects of GAD and violence exposure on MDD, future research should be devoted to elucidating the underlying factors of this phenomenon.

## Figures and Tables

**Table 1:** Demographic Comparisons (N = 593).

	MDD N = 323	No-MDD N = 270	Statistical Comparison
**Age**			
Mother’s Age (Years)	33.6 (9.8)	37.0 (10.3)	*p* < 0.001, d = 0.34
Newborn’s Age (Months)	8.0 (4.1)	9.0 (3.8)	*p* = 0.005, d = 0.24
**Race/Ethnicity**			
Asian or Pacific Islander	8.7% (28)	9.6% (26)	*p* = 0.021 CC = 0.138
Black	21.7% (70)	14.1% (38)	
Hispanic or Latino/a	19.8% (64)	14.4% (39)	
White	45.5% (147)	55.2% (149)	
Another Race or Ethnicity	4.3% (14)	6.7% (18)	
**Marital Status**			
Married	51.9% (167)	57.5% (154)	*p* = 0.023 CC = 0.137
Never Married	36.0% (116)	26.1% (70)	
Separated	5.6% (18)	4.5% (12)	
Divorced	4.3% (14)	9.3% (25)	
Widowed	2.2% (7)	2.6% (7)	
**Education Level**			
No High School Diploma	6.2% (20)	4.1% (11)	*p* = 0.392
High School Diploma/GED	25.1% (81)	22.2% (60)	
Some College	17.3% (56)	21.9% (59)	
Associate’s Degree	11.1% (36)	14.1% (38)	
Bachelor’s Degree	20.7% (67)	21.5% (58)	
Graduate/Professional Degree	19.5% (63)	16.3% (44)	
**Employment Status**			
Full-time	54.8% (177)	48.9% (131)	*p* = 0.040 CC = 0.139
Part-time	18.6% (60)	15.7% (42)	
Unemployed, looking for work	15.8% (51)	14.9% (40)	
Student, Retired, Disabled	4.3% (14)	6.3% (17)	
Stay-at-Home-Mom, Homemaker	5.0% (16)	10.1% (27)	
Other	1.5% (5)	4.1% (11)	
**Healthcare Coverage**			
Employer-Based	29.8% (96)	41.5% (112)	*p* = 0.015 CC = 0.153
Private-Pay/Healthcare.gov	12.4% (40)	14.8% (40)	
Medicaid/CHIP/Government	28.9% (93)	25.2% (68)	
Medicare	19.3% (62)	11.1% (30)	
Other type (Military, etc.)	4.0% (13)	3.0% (8)	*p* = 0.575 CC = 0.023
No Insurance Coverage	5.6% (18)	4.4% (12)	
**First Child or Other Children**			
**First Child**	8.7% (28)	7.4% (20)	
**Other Children**	91.3% (295)	92.6% (250)	

**Table 2:** Psychosocial Factors Comparisons.

	MDD N = 323	No-MDD N = 270	Statistical Comparison
**Generalized Anxiety** (GAD-7)			
	61.1% (196)	9.3% (25)	*p* < 0.001; CC = 0.470
**Suicide Ideation** (PHQ-9, Item 9 *)			
Not at all	19.2% (61)	87.0% (234)	
Several days	24.3% (77)	11.5% (31)	
More than half the days	37.2% (118)	0.7% (2)	
Nearly every day	19.2% (61)	0.7% (2)	*p* < 0.001; CC = 0.573
**Perceived Stress** (PSS)			
	22.0 (4.7)	15.8 (5.8)	*p* < 0.001; d = 1.17
**Violence Screener** (BRFSS)			
	52.0% (168)	17.0% (46)	*p* < 0.001; CC = 0.341
**Social Support** (ISEL-12)			
Total ISEL-12 Score	30.4 (5.4)	35.8 (7.8)	*p* < 0.001; d = 0.82

* PHQ-9 Item 9 states: “In past 2 weeks, how often have you thought… you’d be better off dead or about hurting yourself?”.

**Table 3:** Substance Use Behaviors Comparisons.

	MDD N = 323	No-MDD N = 270	Statistical Comparison
**Alcohol Use/Abuse**			
Total Score	10.9 (9.5)	4.2 (5.7)	*p* < 0.001; d = 0.84 CC = 0.354
Abstainer	17.3% (53)	26.8% (64)	
Low Risk Consumption	28.1% (86)	54.4% (130)	
Harmful Consumption	18.3% (56)	10.5% (25)	
Moderate-Severe Alcohol Use Disorder	36.3% (111)	8.4% (20)	
**Drug Use/Abuse**			
Total Score	5.1 (5.1)	1.8 (3.2)	*p* < 0.001; d = 0.76 CC = 0.367
None	19.1% (53)	43.4% (106)	
Low Risk	42.6% (118)	49.2% (120)	
Moderate Risk	18.4% (51)	3.7% (9)	
Substantial Risk	15.9% (44)	1.6% (4)	
Severe Risk	4.0% (11)	2.0% (5)	

**Table 4:** Hierarchical Binary Logistic Regression—Predicting Major Depressive Disorder (MDD).

	Block 1	Block 2	Block 3
**Age**	0.969 [0.949, 0.989] *p* = 0.003	0.985 [0.958, 1.013] *p* = 0.303	0.981 [0.953, 1.010] *p* = 0.190
**Race/Ethnicity** (Ref: Not White)	0.790 [0.531, 1.177] *p* = 0.247	0.656 [0.379, 1.137] *p* = 0.133	0.679 [0.387, 1.192] *p* = 0.178
**Marital Status** (Ref: Not Partnered)	0.835 [0.564, 1.234] *p* = 0.365	0.981 [0.572, 1.682] *p* = 0.943	1.145 [0.657, 1.996] *p* = 0.632
**Employment Status** (Ref: Not Working)	1.487 [0.967, 2.286] *p* = 0.071	1.687 [0.910, 3.128] *p* = 0.097	1.899 [1.009, 3.574] *p* = 0.047
**Anxiety Disorder** (Ref: No GAD)		5.692 [3.068, 10.562] *p* < 0.001	5.694 [3.024, 10.725] *p* < 0.001
**Perceived Stress Score** (PSS)		1.238 [1.156, 1.326] *p* < 0.001	1.205 [1.124, 1.292] *p* < 0.001
**Violence Screener** (Ref: None)		2.184 [1.166, 4.091] *p* = 0.015	2.278 [1.201, 4.321] *p* = 0.012
**Alcohol Use Score** (AUDIT)		1.063 [1.018, 1.109] *p* = 0.006	1.050 [1.005, 1.096] *p* = 0.029
**Drug Use Score** (DAST)		1.106 [1.028, 1.190] *p* = 0.007	1.088 [1.012, 1.171] *p* = 0.023
**Social Support Score** (ISEL-12)			0.926 [0.884, 0.970] *p* = 0.001
**Constant**	*p* = 0.004	*p* < 0.001	*p* = 0.110
**Omnibus Test**	X^2^(4) = 21.813 *p* < 0.001	X^2^(9) = 275.906 *p* < 0.001	X^2^(10) = 286.895 *p* < 0.001

Note: Values shown are Odds Ratios, 95% Confidence Intervals, and *p*-values. Block 1 including covariates; Block 2 including Psychosocial Factors and Substance Use Measures; Block 3 including Social Support (ISEL).

## Data Availability

Data are available upon request to the corresponding author.

## References

[1] Fawcett E.J., Fairbrother N., Cox M.L., White I.R., Fawcett J.M. (2019). The Prevalence of Anxiety Disorders during Pregnancy and the Postpartum Period: A Multivariate Bayesian Meta-Analysis. J. Clin. Psychiatry.

[2] Howard L.M., Ryan E.G., Trevillion K., Anderson F., Bick D., Bye A., Byford S., O’Connor S., Sands P., Demilew J. (2018). Accuracy of the Whooley questions and the Edinburgh Postnatal Depression Scale in identifying depression and other mental disorders in early pregnancy. Br. J. Psychiatry.

[3] Beck C.T., Records K., Rice M. (2006). Further development of the Postpartum Depression Predictors Inventory-Revised. J. Obstet. Gynecol. Neonatal. Nurs..

[4] Centers for Disease Control and Prevention Pregnancy-Related Deaths: Data from 14 U.S. Maternal Mortality Review Committees, 2008–2017. https://www.cdc.gov/reproductivehealth/maternal-mortality/erase-mm/mmr-data-brief.html.

[5] Avital O., Schub T., Hanson D. (2021). Postpartum Depression. CINAHL Nursing Guide.

[6] Uwadiale A., Cordaro M., Brunett K., Stern M., Howard K. (2022). Lessons Learned about the Need for Early Screening for Depression during the First Months of the COVID-19 Pandemic in the United States. Issues Ment. Health Nurs..

[7] Hasin D.S., Sarvet A.L., Meyers J.L., Saha T.D., Ruan W.J., Stohl M., Grant B.F. (2018). Epidemiology of Adult DSM-5 Major Depressive Disorder and Its Specifiers in the United States. JAMA Psychiatry.

[8] Cordaro M., Grigsby T.J., Howard J.T., Deason R.G., Haskard-Zolnierek K., Howard K. (2021). Pandemic-Specific Factors Related to Generalized Anxiety Disorder during the Initial COVID-19 Protocols in the United States. Issues Ment. Health Nurs..

[9] Ruscio A.M., Hallion L.S., Lim C.C.W., Aguilar-Gaxiola S., Al-Hamzawi A., Alonso J., Andrade L.H., Borges G., Bromet E.J., Bunting B. (2017). Cross-sectional Comparison of the Epidemiology of DSM-5 Generalized Anxiety Disorder Across the Globe. JAMA Psychiatry.

[10] Safi-Keykaleh M., Aliakbari F., Safarpour H., Safari M., Tahernejad A., Sheikhbardsiri H., Sahebi A. (2022). Prevalence of postpartum depression in women amid the COVID-19 pandemic: A systematic review and meta-analysis. Int. J. Gynaecol. Obstet..

[11] Acuff S.F., Strickland J.C., Tucker J.A., Murphy J.G. (2022). Changes in alcohol use during COVID-19 and associations with contextual and individual difference variables: A systematic review and meta-analysis. Psychol. Addict. Behav..

[12] Schmidt R.A., Genois R., Jin J., Vigo D., Rehm J., Rush B. (2021). The early impact of COVID-19 on the incidence, prevalence, and severity of alcohol use and other drugs: A systematic review. Drug Alcohol. Depend..

[13] White A.M., Castle I.-J.P., Powell P.A., Hingson R.W., Koob G.F. (2022). Alcohol-Related Deaths during the COVID-19 Pandemic. JAMA.

[14] Cartus A.R., Li Y., Macmadu A., Goedel W.C., Allen B., Cerdá M., Marshall B.D.L. (2022). Forecasted and Observed Drug Overdose Deaths in the US during the COVID-19 Pandemic in 2020. JAMA Netw. Open.

[15] Howard J.T., Sparks C.S., Santos-Lozada A.R., Olowolaju S.A., Janak J.C., Howard K.J. (2021). Trends in Mortality Among Pregnant and Recently Pregnant Women in the US, 2015–2019. JAMA.

[16] Howard J.T., Perrotte J.K., Flores K., Leong C., Nocito J.D., Howard K.J. (2022). Trends in Binge Drinking and Heavy Alcohol Consumption among Pregnant Women in the US, 2011 to 2020. JAMA Netw. Open.

[17] Almeida M., Shrestha A.D., Stojanac D., Miller L.J. (2020). The impact of the COVID-19 pandemic on women’s mental health. Arch. Women’s Ment. Health.

[18] Nomura Y., Kittler P., Taveras S., Sie S.Y., Nelson E., Davey K., Kaushal R., Rajendran K., Gordon A., Shereen D. (2021). Psychological Effects of COVID-19 on Pregnant Women and New Mothers Living in a US Hotspot. https://europepmc.org/article/ppr/ppr368725.

[19] Qiu J., Shen B., Zhao M., Wang Z., Xie B., Xu Y. (2020). A nationwide survey of psychological distress among Chinese people in the COVID-19 epidemic: Implications and policy recommendations. Gen. Psychiatry.

[20] Shuman C.J., Peahl A.F., Pareddy N., Morgan M.E., Chiangong J., Veliz P.T., Dalton V.K. (2022). Postpartum depression and associated risk factors during the COVID-19 pandemic. BMC Res. Notes.

[21] Wisner K.L., Sit D.K.Y., McShea M.C., Rizzo D.M., Zoretich R.A., Hughes C.L., Eng H.F., Luther J.F., Wisniewski S.R., Costantino M.L. (2013). Onset Timing, Thoughts of Self-harm, and Diagnoses in Postpartum Women with Screen-Positive Depression Findings. JAMA Psychiatry.

[22] Smith C.L., Waters S.F., Spellacy D., Burduli E., Brooks O., Carty C.L., Ranjo S., McPherson S., Barbosa-Leiker C. (2021). Substance use and mental health in pregnant women during the COVID-19 pandemic. J. Reprod. Infant Psychol..

[23] Joyce K.M., Cameron E.E., Sulymka J., Protudjer J.L.P., Roos L.E. (2022). Changes in Maternal Substance Use during the COVID-19 Pandemic. J. Stud. Alcohol. Drugs.

[24] Camden A., To T., Ray J.G., Gomes T., Bai L., Guttmann A. (2022). Categorization of Opioid Use among Pregnant People and Association with Overdose or Death. JAMA Netw. Open.

[25] Swendsen J.D., Merikangas K.R. (2000). The comorbidity of depression and substance use disorders. Clin. Psychol. Rev..

[26] Forray A., Yonkers K.A. (2021). The Collision of Mental Health, Substance Use Disorder, and Suicide. Obstet. Gynecol..

[27] Rutman D., Hubberstey C., Poole N., Schmidt R.A., Van Bibber M. (2020). Multi-service prevention programs for pregnant and parenting women with substance use and multiple vulnerabilities: Program structure and clients’ perspectives on wraparound programming. BMC Pregnancy Childbirth.

[28] Evans M.L., Lindauer M., Farrell M.E. (2020). A Pandemic within a Pandemic—Intimate Partner Violence during COVID-19. N. Engl. J. Med..

[29] Da Thi Tran T., Murray L., Van Vo T. (2022). Intimate partner violence during pregnancy and maternal and child health outcomes: A scoping review of the literature from low-and-middle income countries from 2016–2021. BMC Pregnancy Childbirth.

[30] Lin N., Woelfel M.W., Light S.C. (1985). The buffering effect of social support subsequent to an important life event. J. Health Soc. Behav..

[31] Kuo C., Schonbrun Y.C., Zlotnick C., Bates N., Todorova R., Kao J.C., Johnson J. (2013). A qualitative study of treatment needs among pregnant and postpartum women with substance use and depression. Subst. Use Misuse.

[32] Goodman D.J., Saunders E.C., Wolff K.B. (2020). In their own words: A qualitative study of factors promoting resilience and recovery among postpartum women with opioid use disorders. BMC Pregnancy Childbirth.

[33] Kroenke K., Spitzer R.L., Williams J.B. (2001). The PHQ-9: Validity of a brief depression severity measure. J. Gen. Intern. Med..

[34] Levis B., Benedetti A., Thombs B.D. (2019). Accuracy of Patient Health Questionnaire-9 (PHQ-9) for screening to detect major depression: Individual participant data meta-analysis. BMJ.

[35] Kim S., Lee H.K., Lee K. (2021). Which PHQ-9 Items Can Effectively Screen for Suicide? Machine Learning Approaches. Int. J. Environ. Res. Public Health.

[36] Cohen S., Hoberman H.M. (1983). Positive Events and Social Supports as Buffers of Life Change Stress1. J. Appl. Soc. Psychol..

[37] Cohen S., Kamarck T., Mermelstein R. (1983). A global measure of perceived stress. J. Health Soc. Behav..

[38] Koziol-McLain J., Coates C.J., Lowenstein S.R. (2001). Predictive validity of a screen for partner violence against women. Am. J. Prev. Med..

[39] Saunders J.B., Aasland O.G., Babor T.F., de la Fuente J.R., Grant M. (1993). Development of the Alcohol Use Disorders Identification Test (AUDIT): WHO Collaborative Project on Early Detection of Persons with Harmful Alcohol Consumption—II. Addiction.

[40] Shevlin M., Smith G.W. (2007). The factor structure and concurrent validity of the alcohol use disorder identification test based on a nationally representative UK sample. Alcohol Alcohol..

[41] Skinner H.A. (1982). The drug abuse screening test. Addict. Behav..

[42] Skinner H.A., Goldberg A.E. (1986). Evidence for a drug dependence syndrome among narcotic users. Br. J. Addict..

[43] Gildner T.E., Uwizeye G., Milner R.L., Alston G.C., Thayer Z.M. (2021). Associations between postpartum depression and assistance with household tasks and childcare during the COVID-19 pandemic: Evidence from American mothers. BMC Pregnancy Childbirth.

[44] Waschmann M., Rosen K., Gievers L., Hildebrand A., Laird A., Khaki S. (2022). Evaluating the Impact of the COVID-19 Pandemic on Postpartum Depression. J. Women’s Health.

[45] Chen Q., Li W., Xiong J., Zheng X. (2022). Prevalence and Risk Factors Associated with Postpartum Depression during the COVID-19 Pandemic: A Literature Review and Meta-Analysis. Int. J. Environ. Res. Public Health.

[46] Santos I.S., Tavares B.F., Munhoz T.N., Manzolli P., de Ávila G.B., Jannke E., Matijasevich A. (2016). Patient health questionnaire-9 versus Edinburgh postnatal depression scale in screening for major depressive episodes: A cross-sectional population-based study. BMC Res. Notes.

[47] Turner S., Mota N., Bolton J., Sareen J. (2018). Self-medication with alcohol or drugs for mood and anxiety disorders: A narrative review of the epidemiological literature. Depress. Anxiety.

[48] Chapman S.L., Wu L.T. (2013). Postpartum substance use and depressive symptoms: A review. Women Health.

[49] Shorey S., Chan S.W., Chong Y.S., He H.G. (2014). Maternal parental self-efficacy in newborn care and social support needs in Singapore: A correlational study. J. Clin. Nurs..

[50] Ujhelyi Gomez K., Goodwin L., Jackson L., Jones A., Chisholm A., Rose A.K. (2021). Are psychosocial interventions effective in reducing alcohol consumption during pregnancy and motherhood? A systematic review and meta-analysis. Addiction.

[51] Schwab-Reese L.M., Schafer E.J., Ashida S. (2017). Associations of social support and stress with postpartum maternal mental health symptoms: Main effects, moderation, and mediation. Women Health.

[52] Monistrol-Mula A., Felez-Nobrega M., Domènech-Abella J., Mortier P., Cristóbal-Narváez P., Vilagut G., Olaya B., Ferrer M., Gabarrell-Pascuet A., Alonso J. (2022). The impact of COVID-related perceived stress and social support on generalized anxiety and major depressive disorders: Moderating effects of pre-pandemic mental disorders. Ann. Gen. Psychiatry.

[53] Feinberg E., Declercq E., Lee A., Belanoff C. (2022). The Relationship between Social Support and Postnatal Anxiety and Depression: Results from the Listening to Mothers in California Survey. Women’s Health Issues.

[54] Leahy-Warren P., McCarthy G., Corcoran P. (2011). Postnatal depression in first-time mothers: Prevalence and relationships between functional and structural social support at 6 and 12 weeks postpartum. Arch. Psychiatr. Nurs..

[55] Ankerstjerne L.B.S., Laizer S.N., Andreasen K., Normann A.K., Wu C., Linde D.S., Rasch V. (2022). Landscaping the evidence of intimate partner violence and postpartum depression: A systematic review. BMJ Open.

[56] Beeble M.L., Bybee D., Sullivan C.M., Adams A.E. (2009). Main, mediating, and moderating effects of social support on the well-being of survivors of intimate partner violence across 2 years. J. Consult. Clin. Psychol..

[57] Krumpal I. (2013). Determinants of social desirability bias in sensitive surveys: A literature review. Qual. Quant..

[58] United States Census Bureau, Fertility Rates: Declined for Younger Women, Increased for Older Women. https://www.census.gov/library/stories/2022/04/fertility-rates-declined-for-younger-women-increased-for-older-women.html..

